# Can stress response genes be used to improve the symbiotic performance of rhizobia?

**DOI:** 10.3934/microbiol.2017.3.365

**Published:** 2017-05-26

**Authors:** José Rodrigo da-Silva, Ana Alexandre, Clarisse Brígido, Solange Oliveira

**Affiliations:** Laboratório de Microbiologia do Solo, Instituto de Ciências Agrárias e Ambientais Mediterrânicas (ICAAM), Instituto de Investigação e Formação Avançada (IIFA), Universidade de Évora, Apartado 94, 7002-554 Évora, Portugal

**Keywords:** biological nitrogen fixation, rhizobia, legume, stress response, symbiotic effectiveness, improvement, chaperone

## Abstract

Rhizobia are soil bacteria able to form symbioses with legumes and fix atmospheric nitrogen, converting it into a form that can be assimilated by the plant. The biological nitrogen fixation is a possible strategy to reduce the environmental pollution caused by the use of chemical N-fertilizers in agricultural fields. Successful colonization of the host root by free-living rhizobia requires that these bacteria are able to deal with adverse conditions in the soil, in addition to stresses that may occur in their endosymbiotic life inside the root nodules. Stress response genes, such as *otsAB*, *groEL*, *clpB*, *rpoH* play an important role in tolerance of free-living rhizobia to different environmental conditions and some of these genes have been shown to be involved in the symbiosis. This review will focus on stress response genes that have been reported to improve the symbiotic performance of rhizobia with their host plants. For example, chickpea plants inoculated with a *Mesorhizobium* strain modified with extra copies of the *groEL* gene showed a symbiotic effectiveness approximately 1.5 fold higher than plants inoculated with the wild-type strain. Despite these promising results, more studies are required to obtain highly efficient and tolerant rhizobia strains, suitable for different edaphoclimatic conditions, to be used as field inoculants.

## 1. Rhizobia-legume Symbiosis

Nitrogen is a macronutrient usually available to plants through mineralization of organic matter [1],[2]. In order to guarantee healthy plant growth, adequate nitrogen levels can be provided as synthetic fertilizers, however its application is expensive, as large fossil energy is required for their production. Furthermore, these fertilizers are prone to leaching and may contaminate the groundwater [3],[4],[5]. Some soil bacteria, designated rhizobia, can establish symbiotic associations with legume plants, and fix atmospheric nitrogen, converting it into an assimilable form to the plant. In exchange, the legume plants provide carbohydrates from the photosynthesis process to rhizobia. This process, known as biological nitrogen fixation (BNF), can reduce the use of chemical N-fertilizers and thus decrease the environmental pollution [6] and contribute for more sustainable agricultural practices.

Rhizobia can live either in the soil as free-living bacteria or within the root nodules of host legumes. Within the nodules, rhizobia convert atmospheric dinitrogen (N_2_) into ammonia as a result of the nitrogenase enzyme complex activity in an ATP-dependent manner. Ammonia can be assimilated by the host, resulting in improved plant growth and productivity [7]. The pairing rhizobia-legume host occurs after a complex molecular crosstalk between both partners [8],[9], which often requires cell-to-cell communication and which is determined by the host specificity. This means that the entry into root cells requires appropriate recognition of specific chemical signals by the host plant, namely the rhizobial Nod factor signaling molecule [6]. The recognition of these molecules triggers the curling of root hairs, allowing the entry of rhizobia in the plant [6]. Rhizobia then form an infection thread, which is an invagination of the plant membrane at the infection focus of the root hair. Through the deformed root hair, these bacteria enter the host plant cells and grow down to the cortical cell layers into the nodule meristem [10]. In some cases, these bacteria enter the root by crack entry , i.e. insert themselves in cracks on the root cells [10]. Rhizobia can also enter the root through epidermal intercellular spaces. A successful infection process ends with the formation of the nodule [11], which begins with the reinitiation of cell division in the root cortex, where rhizobial cells will be allocated and will initiate nitrogen fixation in exchange for carbon from the legume host [6].

In terms of taxonomy, rhizobia are currently assigned to 14 different genera. Most of them, including the agriculturally important nitrogen-fixing genera, belong to the *Alphaproteobacteria* class. Only a few genera belong to the *Betaproteobacteria* class [12],[13],[14] (http://www.rhizobia.co.nz/taxonomy/rhizobia). There are presently 89 genomes completely sequenced and annotated, including different strains and symbiovars from the same species. The size of most rhizobial genomes ranges between 6.5 and 9 Mb, and may include plasmids larger than 2 Mb [15].

Rhizobia genomes include two major components: the core genome (higher GC content) that comprises the housekeeping genes, which are responsible for the functioning of the cell, as well as other genes also involved in its essential maintenance [16], and the accessory genome, which is located on plasmids or chromosomal islands (lower GC) and is composed of genes that confer special characteristics to these organisms, such as antibiotics resistance and symbiosis genes [17],[18].

There are two main groups of genes responsible for the symbiosis process in rhizobia, namely genes involved in the nodulation process and those responsible for nitrogen fixation [19]. Nodulation genes (e.g. *nodABC*) encode enzymes responsible for the biosynthesis and secretion of Nod factors, which are lipochitooligosaccharides (LCOs) that interact with plant flavonoids, thus important for determining the rhizobium-legume pairing [6],[20]. Different rhizobia species can have different *nod* genes, therefore producing LCOs with varied structures [21]. Genes involved in the nitrogen fixation process include those that encode the nitrogenase enzyme (*nifHDK*), responsible for the capture and conversion of atmospheric nitrogen into ammonia [22]. In addition to these major groups of genes, there are many others with important functions, both in nodulation and nitrogen fixation. For example, *nodPQ*, *nodX*, *nodEF* and *noe* genes are involved in the synthesis of Nod factors substituents, while *nifA*, *fixLJ*, *fixK* encode transcriptional regulators and *fixABCX* are involved in the electron transport chain to nitrogenase [19].

Besides the symbiosis genes, which are crucial for the interaction with the legume host, rhizobia genomes harbor other genes important for the rhizobia lifestyle, such as stress response genes that allow these bacteria to survive in the soil challenging conditions as well as inside the root nodules.

## 2. Stress Response Genes

Bacteria are not only able of colonizing extreme environments, but also of living inside a wide diversity of hosts. Regardless the particular natural environment where each species can be found, bacteria are often subject to adverse conditions. The main factors studied as bacterial stressors are temperature, salt, pH and nutrient starvation. Many of the genes involved in stress response are conserved across bacterial species, which is remarkable, taking into account the range of different environmental niches where bacteria can live.

Survival under non-optimal conditions requires, first of all, the ability to sense these fluctuations in the immediate surroundings and secondly, the ability to modulate gene expression in order to adjust bacterial physiology to new conditions. Sensing an extracellular change might involve periplasmic protein sensors, as for example sensing envelope stress including pH or salt [23], or *cis*-acting RNA elements, as in the case of temperature-sensing [24]. A rapid and efficient way of modulating gene expression is the regulation of a particular class of transcriptional regulators called sigma factors. Sigma factors are needed for transcription initiation and allow differential gene expression by targeting the RNA polymerase to specific promoters [25]. For example, in *Escherichia coli*, σ^70^ (or RpoD) is the housekeeping sigma factor, while σ^32^ (or RpoH) regulates the heat shock response [26].

Analysis of bacterial response to a given stressor allows the identification of genes that are differentially regulated in response to that stimulus. Global response analyses, such as transcriptomics or proteomics studies, provide important overall maps of the alterations at transcriptional and translational level, respectively.

The heat shock response is particularly well studied in many bacteria [27], including rhizobia. The heat shock proteins (HSPs) are encoded by genes induced after a sudden increase of temperature. There are two major classes of HSPs involved in protecting cells from protein denaturation caused by temperature upshift: chaperones and proteases. Chaperones systems, such as GroESL and DnaKJ, have the important role of rescuing misfolded proteins and allowing their refolding into the native and functional conformation. Proteases, as for example FtsH and ClpXP, are involved in the degradation of protein aggregates (misfolded proteins that are no longer able to acquire their native conformation). It is noteworthy that these HSPs are often involved in the response to other stressors and moreover, chaperones and proteases are also important under normal conditions, namely for the correct folding of newly synthesized polypeptides.

### 2.1. Molecular bases of stress response in rhizobia

The study of the molecular bases of stress response in rhizobia is particular interesting since these bacteria are exposed not only to the soil conditions, but also to the endosymbiotic lifestyle, inside the host plant root or shoot nodules. On a more applied perspective, development of highly effective rhizobia strains to be used as field inoculants must not disregard the importance of stress tolerance. If the inoculant formulation is not able to survive to abiotic stresses, its successful performance in the field is greatly compromised.

Transcriptomic studies have given an important contribution to understand the global response of these large genome bacteria to stress conditions. Different rhizobia genera nodulating distinct hosts have been studied and although some common trends can be identified, the transcriptional profile of the response to the same stressor is diverse among rhizobia.

Soil salinity is an important problem affecting soils worldwide, particularly in developed countries where irrigation is a common agricultural practice [28]. Some legume species are able to grow under moderate salinity conditions and effectively increase the available N [29]; however, their ability to establish nitrogen-fixing symbiosis relies on the tolerance of compatible rhizobia to the same stress. A recent study on the response to salt shock of *Mesorhizobium* strain MAFF303099 (currently reclassified as *Mesorhizobium japonicum* [30]), analysed the transcriptional response of this strain and compared it to other rhizobia previously studied, from different species and hosts [31]. Contrary to most studies, which report the induction of genes involved in the synthesis of osmoprotectant molecules, as for example trehalose [32]–[35], *M. japonicum* response to salinity did not include changes in the transcription of those genes. In addition, no sigma factor showed to be transcriptionally regulated by salinity in *M. japonicum*, while both *Sinorhizobium meliloti* and *Rhizobium etli* showed the upregulation of *rpoH2*, among others.

In terms of heat shock response, similarly to other bacteria, different rhizobial species showed, as expected, the induction of genes encoding chaperones and other HSPs [15]. A study on *M. japonicum* MAFF303099 showed that from the genes differentially expressed following heat shock, a large proportion was underexpressed [36], while in *S. meliloti* 1021 and *R. etli* CE3 the number of genes over- and underexpressed was approximately the same [32],[37]. Another study with *S. meliloti* 1021 also showed more genes underexpressed than overexpressed in response to heat shock [38], yet in much lower proportion than for *M. japonicum*.

Global response to acid pH remains less studied, nevertheless the available reports indicate, once again, that different rhizobia may show little similarities on their acid transcriptional profile [39],[40],[41]. While in *S. meliloti*, exposure to acid pH lead to a strong upregulation of genes involved in exopolysaccharide biosynthesis as well as a general downregulation of genes related to motility and chemotaxis [40], in *M. japonicum* these genes remained mostly unchanged [39]. In both strains, genes involved in ABC transporter systems were overexpressed, with higher numbers in the case of *M. japonicum*. More recently a multi-omics approach was carried out to fully characterize the response to acid stress of *S. meliloti* [42]. This comprehensive analysis showed that acid adaptation requires cell envelope remodelling and that under controlled acidic conditions, *S. meliloti* increases aerobic respiration and alters the central carbon metabolism.

In general, when different stresses are compared using the same strain, most of the genes differentially expressed are stress-specific. Furthermore, contribution of each replicon to different stresses also varies. For example, from the six plasmids included in the *R. etli* CE3 genome, four were found to be highly over-represented in the response to heat shock, while most genes overexpressed after a saline shock were chromosomal and encoded in a fifth different plasmid [32]. In addition, stress response may also differ in similar organisms with different levels of tolerance to the imposed stress [39]. These global transcriptional analyses (using microarrays or RNAseq) also demonstrate that there is still a high percentage of genes of unknown function, whose expression responds to environmental perturbations, and that might have an important role on survival to stress.

Functional studies that focus on a given stress response gene or operon also represent important contributions to our understanding of the molecular bases of stress response. Rhizobial genomes typically encode several copies of the major chaperone system GroESL and these genes were known to be essential for *E. coli* viability [43] as well as determinant for the limit temperature for growth [44]. Their study in rhizobia showed that there is some functional redundancy among different copies, although different regulatory mechanisms of these operons can be found in the same strain. The differential regulation of these operons allows a fine tune of the GroESL pool under different conditions, including inside the nodule, during symbiosis with the host plant [45]. For example, in *S. meliloti* and *Bradyrhizobium japonicum*, both with five *groEL* copies, all *groEL* single mutants are viable [46],[47]. Contrary to this, one of the three *groEL* copies of *R. leguminosarum* is essential for growth [48]. In terms of heat tolerance phenotype, a *S. meliloti* strain with mutations in *groEL*_1_ and *groEL*_5_ showed a slower growth compared to the wild-type, especially under higher temperatures [47].

Other important chaperone genes such as *dnaK* and *dnaJ* have also been implicated in stress tolerance of several rhizobia species. *dnaJ* mutants in both *B. japonicum* and *R. tropici* showed reduced growth at high temperatures [49],[50]. Furthermore, a comparison of the transcript levels of these chaperone genes between heat-tolerant and heat-sensitive isolates of the same *Mesorhizobium* species indicated that tolerant isolates consistently showed higher levels of *groEL* and *dnaK* transcripts after heat shock [51]. Similarly, acid-tolerant mesorhizobia isolates showed higher levels of these chaperone transcripts when compared to acid-sensitive isolates, suggesting the involvement of these genes in acid tolerance as well [52]. In terms of tolerance to salinity, no correlation was found between the isolates phenotype and the major chaperones transcript levels after a salinity shock [53]. In addition, the characterization of the stress tolerance phenotype of a *Mesorhizobium clpB* mutant indicated the involvement of this chaperone gene in the tolerance to some heat and acidity conditions, but not in the tolerance to salinity [54].

Soil acidity is a widespread problem that is not confined to the effects of H^+^ on crops, since acid soils often present aluminium and manganese toxicity problems as well as low availability of calcium and magnesium [55]. Studies on acid tolerance have provided evidence on genes important to growth and survival in low pH conditions. *act* (acid tolerance) genes were firstly described in *S. meliloti*, in particular *actA*, whose disruption was shown to confer a pH sensitivity phenotype in a Tn5 mutagenesis screening [56]. Besides *act* genes (also *actR/S* and *actP*), other genes were found to be involved in rhizobial acid tolerance, such as *exo* genes, *lpiA* and *phrR* genes [57],[58],[59].

Several studies showed that some genes may be involved in the tolerance to several stress conditions. For example, genes involved in trehalose biosynthesis (*otsAB*, *treS* and *treZY* genes) have been associated to rhizobial tolerance to desiccation, salinity and heat [60],[61]. Besides its action as stress protectant, in free-living rhizobia, trehalose may be used as carbon and energy source. Trehalose has also been detected in bacteroids, however it seems that trehalose synthesis has different pathways under free-living or symbiotic conditions [60].

In agricultural soils, native rhizobia populations might be highly inefficient in promoting the host plant growth, but they are usually well adapted to more adverse conditions in the soil. Therefore, the investigation of the molecular bases of stress tolerance represents a fundamental step towards the improvement of rhizobia inoculants to be used under field conditions.

### 2.2. The role of stress response genes in the symbiosis

The symbiotic process between rhizobia and the host legume is mainly divided into two major events: bacterial infection and nodule organogenesis [11]. For a successful symbiotic association, it is essential that these two phenomena are coordinated in both spatial and temporal manner, to ensure nodule formation at the site of bacterial infection (for review see [11]). When compatible molecular signals are recognized by the host legumes, a series of events, such as growth of polarized root hair tip and invagination associated with bacterial infection, are initiated in the host plant leading to the development of specialized structures, called nodules [62],[63].

Since early events in the symbiosis process such as molecular signalling and rhizobial attachment, are particularly sensitive to high temperatures, salinity, acidity and other environmental stresses [64],[65],[66], rhizobia have to be able to physiologically adapt to environmental conditions, in order to ensure a successful symbiosis with its legume partner. These stresses that negatively affect the microsymbiont in free-living conditions as well as during the symbiotic relationship can lead to a delay in infection and nodule formation, development of non-fixing nodules or even to failure of the nodulation process [67]. Moreover, during infection, rhizobia also have to deal with adverse conditions within the host cells and with the plant innate immunity that induces physiological stress responses, which may interfere with symbiosis [68]. For example, the pH in the rhizosphere of the leguminous host plant is decreased due to protons and organic acids excreted by the plants. The pH is also lower within the plant cell, due to the transport of protons or ionized acids that acidify the symbiosomes [69]. In addition, low oxygen concentration in the nodules, which can alter the pathways of carbon metabolism, leads to the production of organic acids that inhibit the regulation of cytoplasmic pH [70]. Therefore, rhizobia have to be able to overcome stress conditions both outside and within the nodule during symbiosis, to achieve a complete and effective nitrogen-fixing symbiosis. As a consequence, the role of stress response genes must be an important or even fundamental part of the symbiotic process. In fact, it was suggested that among the genes required for bacteroid formation, some are specific for symbiosis and others are involved in physiological adaptation to the environmental conditions within and outside the nodule [71].

Transcriptomic and proteomic analyses of rhizobia in symbiosis with their host legumes suggest the involvement of stress response genes, mainly heat shock proteins such as ClpB and GroESL, in the symbiotic process. For example, overexpression of the ClpB and GroEL/ES proteins was detected in nodules formed by *Bradyrhizobium japonicum* and *Sinorhizobium meliloti* strains [72]–[76]. These findings are reinforced through transcriptomic analyses where up-regulation of these genes was observed in root nodules [77],[78],[79].

Although these approaches provide a global view on putative genes involved in symbiosis, the involvement of a specific gene in the symbiotic process requires other strategies, such as gene knockout. Several studies focused in determining the involvement of stress response genes, mainly heat shock proteins, in the symbiotic process have been performed. The most studied molecular chaperone in terms of its involvement in the symbiosis is GroEL. Particular copies of this chaperone gene, usually upregulated in the bacteroids, seem to play a fundamental role in the formation of functional NodD and nitrogenase complex [80],[81]. For example, among the five *groESL* operons in the *S. meliloti* genome only one operon (*groEL_1_*) was found to be involved in symbiosis [81]. Fischer et al. [46] found a co-regulation between *groESL_3_* and nitrogen fixation genes in *B. japonicum*, yet none of the *B. japonicum* mutants that individually lack one *groEL* gene were depleted in their symbiotic phenotype [47],[80]. However, double mutation on *groEL*_3_ and *groEL*_4_ genes in *B. japonicum* affects the symbiotic performance, since these copies are required for the formation of a functional nitrogenase [80]. These two copies are the most abundant in the GroEL pool in bacteroids [46]. Studies on the symbiotic performance of strains mutated in the *dnaJ* gene also revealed distinct results, using different rhizobia species. For example, a *B. japonicum* *dnaJ* mutant strain was able to establish fully effective symbiosis with soybeans [49]. In contrast, Nogales et al. [50] found that a *dnaJ* mutant of *Rhizobium tropici* was able to form nodules in *Phaseolus vulgaris*, however this mutant showed low nitrogenase activity, which was also evident in the reduced plant growth and in the reduction of the nitrogen content of the plant shoots. Similarly, *dnaJ* is required for effective symbiosis of *R. leguminosarum* bv. *phaseoli* [82]. On the other hand, the DnaK chaperone, another protein that constitutes the DnaK-DnaJ chaperone system, is required for optimum symbiotic function in *S. meliloti* [83]. More recently, the involvement of the ClpB chaperone in the symbiotic process was evaluated. Although a *Mesorhizobium clpB* mutant strain was able to establish symbiosis with chickpea plants, the ClpB absence caused a delay in nodule formation and development [54], indicating its involvement in the symbiotic process.

Other genes involved in stress response, namely major regulators of the heat shock response, have been implicated in the symbiosis. For instance, *S. meliloti* *rpoH1* mutants have been shown to have defective symbiotic phenotypes, showing poor colonization and survival in bacteroids and do not fix nitrogen [84],[85]. In contrast, a *rpoH2* mutant showed a symbiotic phenotype similar to the wild-type [84],[85],[86]. Nevertheless, *rpoH1 rpoH2* double mutants exhibited a more severe symbiotic phenotype than the *rpoH1* mutant [87]. Similar results were obtained by Martinez-Salazar et al. [88] where *R. etli* *rpoH1* and *rpoH2 rpoH1* mutants exhibited reduced nitrogenase activity and bacterial viability in early and late symbiosis, compared with nodules formed by the *rpoH2* mutant and wild-type.

Despite the fact that functional studies showed results that vary with the rhizobia species analysed, stress response genes seem to be implicated in rhizobial infection and nitrogen-fixation. The lower symbiotic performance obtained with most of the chaperone mutants, suggests that the role of chaperones is important for bacterial cells to achieve an efficient and effective symbiotic interaction with their legume hosts. The negative effects in their symbiotic phenotypes, due to the loss of specific chaperone genes, is most likely due to the role of these proteins in the folding of newly synthesized polypeptides, refolding of denatured proteins and disaggregation of proteins involved in the symbiosis. Proteins denaturation and aggregation may occur under the environmental conditions found by rhizobia within the root cells, such as acidity or microaerobic conditions. Accordingly, induction of genes encoding molecular chaperones and proteases in rhizobial cells grown under acidity or microaerobic conditions have been described [40],[77],[89],[90]. Similarly, both *rpoH* genes were induced under microaerobic conditions but only *rpoH1* was overexpressed in heat shock and oxidative conditions, whereas *rpoH2* was induced as response to osmotic conditions [88]. Another regulator, *rpoE4*, is upregulated under oxidative, saline and osmotic stress, and microaerobic conditions [91],[92], and it is also overexpressed in aggregated cells during biofilm formation [93], which is an essential step in the early stages of nodulation [94].

Altogether, the major chaperone genes seem to be involved in the symbiotic process between rhizobia and legume hosts, not only due to their role in the folding of important symbiosis proteins, but also due to their direct involvement in response to stressful conditions found in the rhizosphere and within the root cells. However, further studies are required to elucidate in which step of the symbiotic interaction these and other stress response genes are particularly important.

## 3. Rhizobia Improvement Using Stress Response Genes

The use of biotechnology in agriculture has been growing over the years as a sustainable strategy for increasing animal and vegetable production [95]. Plant growth-promoting bacteria (PGPB) which include rhizobia, can be used as tools to increase the production of crop plants, while reducing the use of environmental damaging chemical fertilizers or pesticides [96]. Rhizobia inoculants should be effective in nitrogen fixation, persistent in soil and competitive with native populations, as well as adapted to the field environmental conditions [97], in order to be able to establish a successful and effective symbiosis.

Molecular biotechnology strategies can contribute to the improvement of rhizobia inoculants, particularly the genetic engineering of rhizobia to overexpress specific genes, directly or indirectly involved in the symbiotic process, in order to improve rhizobia performance such as symbiotic effectiveness, nodulation efficiency, competitiveness and stress tolerance.

Several genes involved in stress response have been overexpressed in rhizobia as an attempt to improve their symbiotic performance particularly under stress conditions such as salinity, oxidative, drought, heat or biotic stress. Overexpression of genes related to protection of bacteria from salt stress has contributed to the improvement of rhizobia strains under stressful conditions. A *S. meliloti* strain overexpressing the *betS* gene, involved in the rapid acquisition of betaines by cells subjected to osmotic shock, showed a better maintenance of nitrogen fixation activity in salinised alfalfa plants than the wild-type strain [98]. The *otsA* gene encodes the enzyme trehalose-6-phosphate synthase involved in the biosynthesis of trehalose [99]. Moussaid et al. [61] overexpressed *otsA* from *S. meliloti* in *Mesorhizobium* *ciceri* and found an increase of the *otsA*-overexpressing strain growth in saline media. Chickpea plants inoculated with *M. ciceri* carrying extra *otsA* copies formed more nodules and accumulated more shoot biomass than the wild-type inoculated plants, when grown in the presence of NaCl. Also, *P. vulgaris* inoculated with *R. etli* overexpressing *otsA* showed more nodules with increased nitrogenase activity and higher biomass compared with plants inoculated with the wild-type strain. Only plants inoculated with the *otsA*-overexpressing strain fully recovered from drought stress [100].

Some enzymes involved in the synthesis of plant hormones may have a role in improving the symbiotic performance of rhizobia when plants are subjected to different types of stresses, both biotic and abiotic stresses. It is the case of 1-aminocyclopropane-1-carboxylate (ACC) deaminase (encoded by the *acdS* gene), which regulates the levels of the ethylene precursor, ACC. Ethylene is produced by plants in response to several environmental stresses and can negatively affect nodulation [101],[102]. *M. ciceri* strains (salt-sensitive and salt-tolerant) transformed with an exogenous *acdS* gene induced a chickpea growth significantly higher, compared with the wild-type strain, in the presence of salt [103]. Furthermore, the *acdS*-transformed salt-sensitive strain was able to induce nodules in the same extent as the salt-tolerant strain under salinity [103]. Kong et al. [104] evaluated the symbiotic performance of a *S. meliloti* strain overproducing ACC deaminase in *Medicago* *lupulina* under copper stress conditions. Plants inoculated with the *acdS*-transformed strain showed higher dry weight, higher total copper uptake but lower levels of copper translocation to aerial parts, as compared with plants inoculated with the wild-type strain. The *acdS* gene was also used by Nascimento et al. [105] to transform a *M. ciceri* strain that was inoculated in chickpea plants growing in non-sterilized soil displaying biotic and abiotic constraints to plant growth. The modified *M. ciceri* strain showed an increased nodulation performance and was able to augment the total biomass of chickpea plants and reduce chickpea root rot disease susceptibility [105]. Similar results were found for chickpea plants inoculated with the same strain under waterlogging conditions [106].

A *S. meliloti* strain harbouring an additional pathway for the synthesis of the plant hormone indole-3-acetic acid (IAA) showed an increased tolerance to several stress conditions such as UV, high salt and low pH [107]. Furthermore, *Medicago* *truncatula* plants inoculated with this strain showed reduced symptoms of senescence, lower expression of ethylene signaling genes, lower reduction of shoot dry weight due to stress, and better nitrogen-fixing capacity [107]. Further studies using this strain also showed that IAA overexpression was responsible for significant increases in both shoot and root fresh weights of *M. truncatula* plants grown under phosphate-starvation [108].

Flavodoxin genes have been used to improve rhizobia performance particularly under oxidative stress. The overexpression of a flavodoxin gene in a *S.* *meliloti* strain was able to protect free-living *S. meliloti* from cadmium toxicity and had a positive effect on nitrogen fixation on alfalfa plants subjected to cadmium stress [109]. In addition, Redondo et al. [110] observed that alfalfa plants inoculated with rhizobia overexpressing flavodoxin displayed a delay in nodule senescence.

It is known that oxygen irreversibly inactivates the rhizobial nitrogenase enzyme [111],[112]. To limit the amount of oxygen in the bacteroids, the legume host synthesizes leghaemoglobin, which has a high affinity for oxygen [112]. As a high amount of oxygen is necessary to supply the energy demands of the nitrogen reduction process, bacteroids produce a high-affinity cytochrome *cbb3*-type oxidase to cope with the low oxygen concentration in the nodule [113]. The inoculation of *P. vulgaris* plants with a *R. etli* strain having enhanced expression of *cbb3* oxidase in bacteroids reduced the sensitivity of *P. vulgaris-R. etli* symbiosis to drought [113]. Others studies have also reported that genetically modified rhizobial strains overproducing *cbb3* oxidase are more efficient in nitrogen fixation under optimal conditions compared to their parental strains [114],[115].

As mentioned previously in section 2.2, several studies have implicated some stress response genes, namely chaperone genes, directly in the symbiotic process. For example, the GroEL-GroES chaperone system, mostly known as important components of the heat shock response [51],[81], seem to be involved in the formation of functional NodD and nitrogenase complex [80],[81]. Similarly, the chaperone ClpB was found to be involved in chickpea root nodulation by *Mesorhizobium* [54]. Recently we have been investigating the use of chaperone genes to improve the symbiotic performance of rhizobia. Mesorhizobia isolates nodulating chickpea, an important legume in human diet and one of the most widely grown pulse crops worldwide, have been used.

The first successful improvement of a rhizobium with a chaperone gene was achieved using the chickpea nodulating strain *M. mediterraneum* UPM-Ca36^T^ modified with extra-copies of the *clpB* gene [116]. The nodulation kinetics analysis showed a higher rate of nodule development as well as a higher number of nodules in plants inoculated with the *clpB*-transformed strain. More interestingly the symbiotic effectiveness of the *clpB*-overproducing strain increased ∼60% at pH 5 and ∼83% at pH 7, compared to the wild-type strain. This improved symbiotic phenotype may be related to an increased expression of symbiosis genes, as detected for the nodulation genes *nodA* and *nodC* [116].

**Figure 1.**
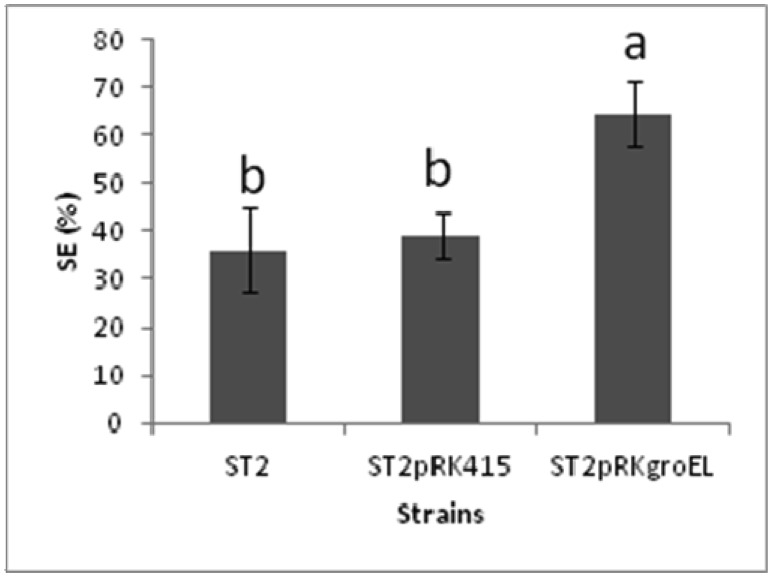
Symbiotic efectiveness (SE) of strains ST2, ST2pRK415 and ST2pRKgroEL inoculated in chickpea plants grown in pots for 7 weeks. Different letters (a and b) correspond to statistically significant differences (*P* < 0.05) detected using the one-way ANOVA and the post hoc Tukey test, implemented in SPSS V.21 software (SPP Inc., Chicago, U.S.A). Bars represent standard deviation.

**Figure 2.**
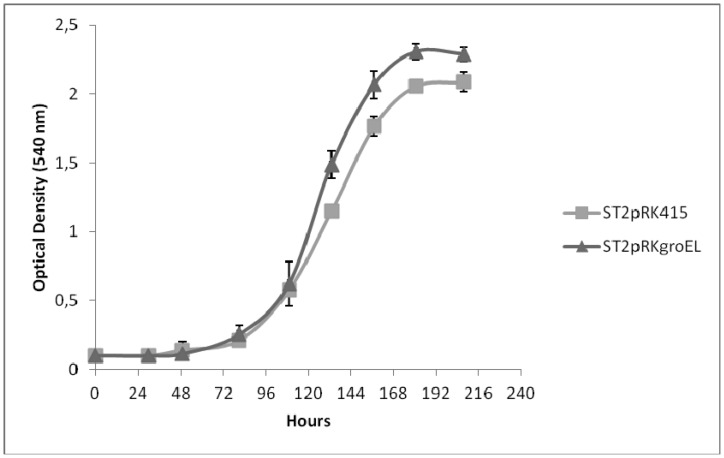
Growth curves of ST2pRK415 and ST2pRKgroEL, after a heat shock at 48 °C during 15 min. There are statistical differences between the growth of the two strains from 133 hours onwards (*P* < 0.05), detected using T-test, implemented in SPSS V.21 software (SPP Inc., Chicago, U.S.A). Bars represent standard deviation.

To investigate the potential of the chaperone gene *groEL* in the improvement of rhizobia symbiotic performance, a chickpea *Mesorhizobium* strain with low symbiotic effectiveness, strain ST-2 [117], was modified by the addition of extra *groEL* copies from *M.* *mediterraneum* UPM-Ca36^T^, cloned in the expression vector pRK415 (ST2pRKgroEL strain). This is the *M. mediterraneum* UPM-Ca36^T^ *groEL* copy that shares the highest similarity with the copy overexpressed in *M. japonicum* MAFF303099 bacteroids [77]. A plant growth trial was carried out for seven weeks in pots filled with sterile vermiculite and the shoot dry weight of inoculated chickpea plants was used to calculate the symbiotic effectiveness (SE) [118]. Figure 1 shows that the SE of ST2pRKgroEL strain is about 1.5 fold higher than the SE of the wild-type or the empty vector strain. This result supports the hypothesis that GroEL, in addition to its main role as heat shock protein, may also be involved in the nitrogen fixation process, thus contributing to increase the symbiotic effectiveness. In *S. meliloti* *groEL* was mutated and affected NodD activity [81], therefore, *groEL* may be involved in the early stages of the symbiosis process. Since GroEL is known to be involved in temperature stress tolerance, the growth of these mesorhizobia strains was evaluated after submitting the modified strains to a heat shock of 48 °C, for 15 minutes and then moved to 28 °C in tryptone-yeast (TY) medium supplemented with 15 µg ml^−1^ tetracycline [119]. Figure 2 shows a higher growth of the strain carrying extra *groEL* copies, compared to the strain carrying the empty plasmid, during late exponential and stationary phases, suggesting that extra copies of the GroEL protein most likely help the bacteria to recover after the heat shock. Nevertheless, the stress response transcriptome analyses of *M. japonicum* MAFF303099 showed that the *groEL* copy found to be induced in bacteroids [77] is not overexpressed in response to heat, acid or salt shocks [36],[39],[120].

Despite the promising results obtained in the improvement of rhizobial symbiotic performance with the overexpression of stress response genes, further studies with more bacterial species and other genes are required to validate this approach as a strategy to engineer rhizobial strains that can be useful as crop inoculants, particularly under challenging soil and climatic conditions.
